# Glycoprotein 130 is associated with adverse postoperative clinical outcomes of patients with late-stage non-metastatic gastric cancer

**DOI:** 10.1038/srep38364

**Published:** 2016-12-05

**Authors:** Yifan Cao, Heng Zhang, Hao Liu, Chao Lin, Ruochen Li, Songyang Wu, Hongyong He, He Li, Jiejie Xu

**Affiliations:** 1 Department of Biochemistry and Molecular Biology, School of Basic Medical Sciences, Fudan University, Shanghai, China; 2 Department of General Surgery, Zhongshan Hospital, Fudan University, Shanghai, China

## Abstract

The interaction of glycoprotein 130 (gp130) with the cytokines of Interleukin-6 (IL-6) family has proved to play a crucial part in several cancers. Our current study is designed to discover the clinical prognostic significance of gp130 in non-metastatic gastric cancer. We examined intratumoral gp130 expression in retrospectively enrolled 370 gastric cancer patients who underwent radical gastrectomy with standard D2 lymphadenectomy at Zhongshan Hospital of Fudan University during 2007 and 2008 by immunohistochemical staining. The expression of gp130 was significantly correlated with T classification, N classification and TNM stage (*P* = 0.003, *P* < 0.001 and *P* < 0.001, respectively; T, N, TNM refers to Tumor Invasion, Regional lymph node metastasis and Tumor Node Metastasis, respectively). Elevated intratumoral gp130 expression implied unfavourable overall survival (OS) (*P* < 0.001) and disease-free survival (DFS) (*P* < 0.001), respectively. Furthermore, among TNM II and III gp130-high patients, those who were treated with 5-fluorouracil (5-FU) based adjuvant chemotherapy had better OS (*P* < 0.001). The generated nomogram performed well in predicting the 3- and 5-year OS of gastric cancer patients. The incorporation of gp130 into contemporary TNM staging system would be of great significance to improve the current individual risk stratification. These findings contribute to better clinical management for those patients who would benefit from adjuvant chemotherapy.

Despite the fact that dramatic decline of gastric cancer incidence has occurred in more developed countries as the United States in the past eighty years^1^, gastric cancer still ranks the second most lethal malignant tumor in less developed countries as China^2^. Growing evidences have proved in recent years that inflammation functions as the direct cause of several malignant tumors^3^,^4^. *Helicobacter Pylori* induced chronic gastritis is generally believed to be a major risk for human gastric cancer, although the comprehensive tumorigenic mechanisms of gastric cancer still remain obscure^5^. Surgical resection is accepted as the sole curative choice for gastric cancer patients, especially for those who suffer from the early stage of the disease^6^. Nevertheless, high risk of disease recurrence in advanced-stage gastric cancer patients pushes clinicians to take postoperative adjuvant treatments into consideration. As a result, 5-fluorouracil (5-FU) based chemotherapy is generally applied as first-line gastric cancer assistant treatment^7^. Unfortunately, the overall survival rates were still far from satisfactory regardless of the fact that the initial rates were high indeed. In this respect, a novel precise stratification for gastric cancer, which can be utilized as a more reliable predictor for patient outcomes and treatment response, is urgently needed.

It is estimated that persistent infection or chronic inflammation could result in at least 20% of cancer incidence. As to the rest 80% which are not derived from inflammation, however, several inflammatory infiltrates that secrete various cytokines are also involved in the tumor microenvironment^8^,^9^,^10^. In gastric cancer, elevated expression of such proinflammatory cytokines as interleukin-6 (IL-6)^11^ and interleukin-11 (IL-11)^12^ have been confirmed the correlation with tumor development.

Glycoprotein 130 (gp130) is a trans-membrane protein which serves as the signaling receptor subunit of the cytokines affiliated to the IL-6 family^13^,^14^. IL-6 and IL-11 are the two dominant cytokines of this family and have proved their existence in gastric mucosa. Additionally, they are the only two IL-6 family cytokines which can exclusively utilize gp130 homodimers^15^. As an inflammation-related cytokine receptor, gp130 plays a crucial part in a series of cancers including ovarian cancer^16^, breast cancer^17^, prostate cancer^18^, colon cancer^19^ and lung cancer^20^. However, the role which gp130 plays in gastric cancer is still unknown and requires further discovery. Consequently, it would be of much necessity to figure out the contribution of gp130 to gastric cancer.

In the present study, we aimed to investigate the potential role of gp130 in the prognostic effect of gastric cancer. Intratumoral gp130 expression was appraised by means of immunohistochemistry and its correlation with clinicopathological characteristics was evaluated. Still further, we demonstrated our hypothesis that the combination of gp130 expression with the current TNM staging system could improve individual risk stratification for gastric cancer to a large extent.

## Results

### Intratumoral gp130 immunohistochemical staining intensity and the correlation with clinicopathological characteristics

**Figure 1:**
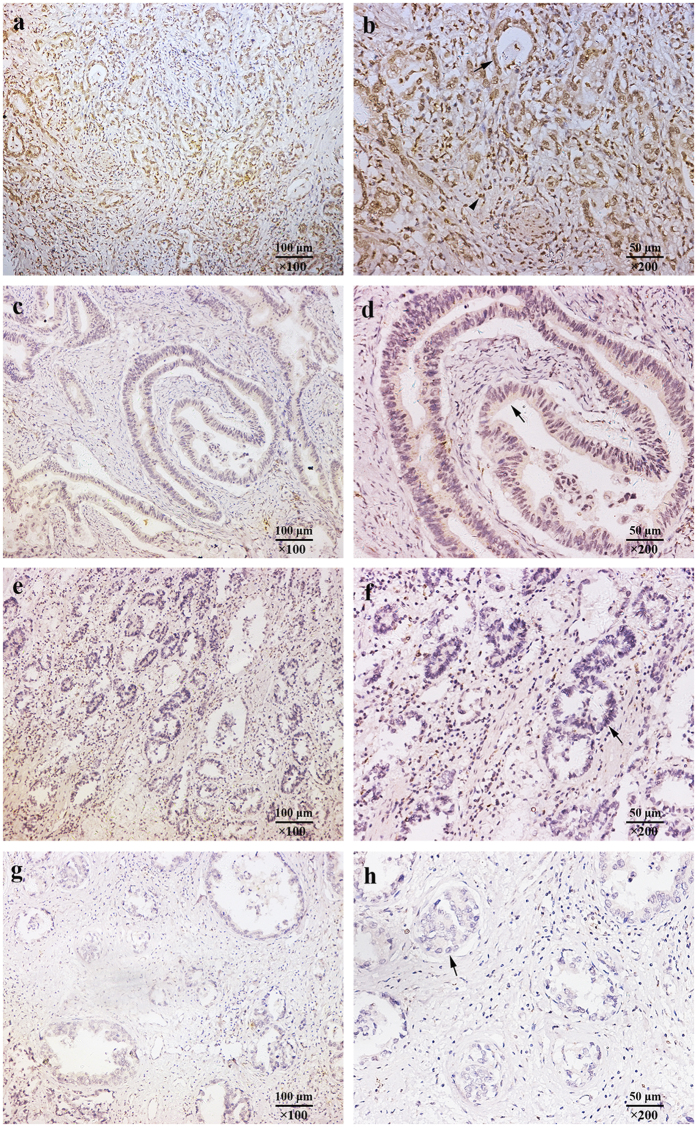
Intratumoral gp130 expression in gastric cancer tissues. (**a**,**b**) Gastric cancer with high intratumoral gp130 expression. Arrow shows gp130-high tumor cells, and arrowhead shows positive stromal cells. (**c**,**d**) Gastric cancer with low intratumoral gp130 expression. Arrow shows gp130-low tumor cells. (**e**,**f**) Gp130-negative gastric cancer tissues. Arrow points at gp130-negative tumor cells. (**g**,**h**) Negative control. Arrow shows tumor cells.

To investigate whether the intratumoral immunohistochemical staining intensity of gp130 is correlated with the development and the progression of gastric cancer, we assessed the intratumoral expression of gp130 by means of IHC staining analysis in the total of 370 non-metastatic gastric cancer patients. Both low and high power representative images for gp130 expression in gastric cancer cells were shown (Fig. 1a–d). Gp130-negative gastric cancer tissues were also observed (Fig. 1e,f). Negative control (Fig. 1g,h) was treated identically but with the primary antibody omitted. The percentage of gp130-high/low patients in different TNM stage was displayed in Supplementary Figure 1a. Western blot analysis showed the specificity of anti-gp130 antibody with use of extracted protein from cultured gastric cancer cell lines (Supplementary Figure 1b). According to the result from western blot, the specificity of anti-gp130 antibody was ensured and thus false positives could be excluded. As to false negatives, the whole set of tumor microarrays (TMA) were immunostained at the same time so as to ensure an objective comparison between different samples. The immunohistochemistry was strictly performed according to the protocol. Negative controls were also performed but with the primary antibody omitted. According to the cut-off value derived from the assessment of immunohistochemical staining intensity, 49.7% (184/370) of the patients recruited were classified as high gp130 expression set. Listed in Table 1 are detailed characteristics of patients. Gp130 expression was significantly associated with T classification (Table 1, *P* = 0.003), N classification (Table 1, *P* < 0.001) and TNM stage (Table 1, *P* < 0.001), respectively.

### Clinical prognostic effect of gp130 on gastric cancer victims

**Figure 2:**
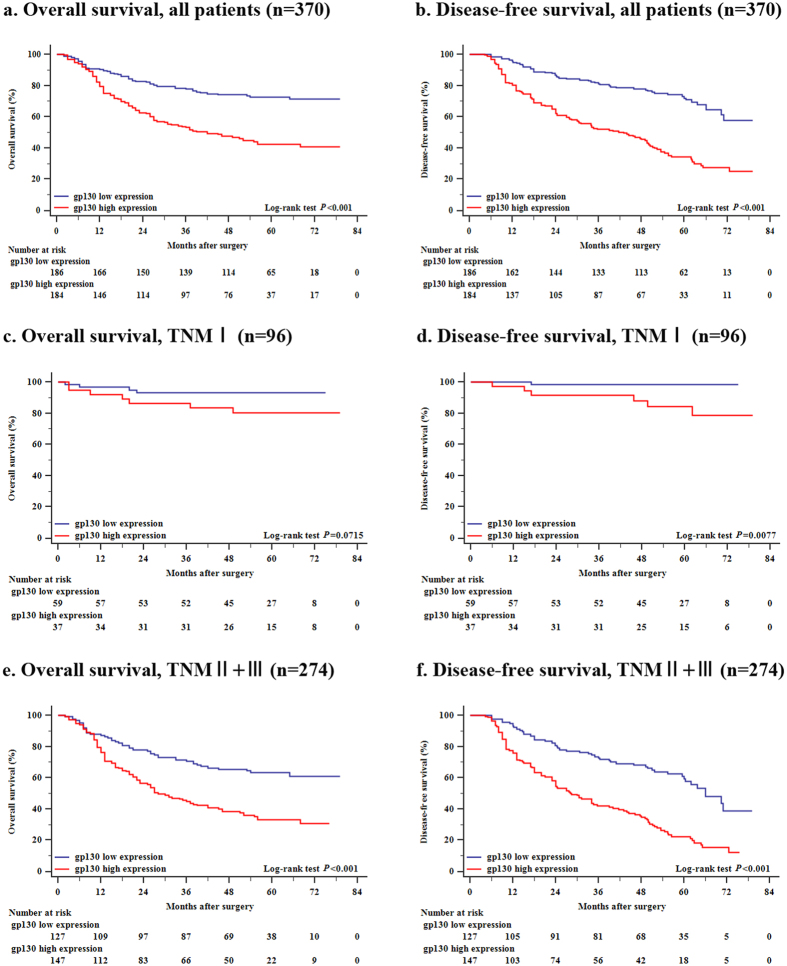
Kaplan–Meier analysis of overall survival (OS) and disease-free survival (DFS) according to gp130 expression in patients with non-metastatic gastric cancer. Kaplan-Meier analysis of overall survival and disease-free survival was applied with respect to gp130 expression in patients with non-metastatic gastric cancer. (**a**) Overall survival, all patients (n = 370, *P* < 0.001). (**b**) Disease-free survival, all patients (n = 370, *P* < 0.001). (**c**) Overall survival, TNM stage I (n = 96, *P* = 0.0715). (**d**) Disease-free survival, TNM stage I (n = 96, *P* = 0.0077). (**e**) Overall survival, TNM stage II + III (n = 274, *P* < 0.001). (**f**) Disease-free survival, TNM stage II + III (n = 274, *P* < 0.001). *P*-values were calculated by means of log-rank test.

As shown in Fig. 2a,b, high expression of gp130 was correlated with both poor overall survival (*P* < 0.001) and disease-free survival (*P* < 0.001) with regard to the whole patient cohort. Furthermore, we combined TNM stage II tumors with TNM stage III tumors as late-stage disease and the TNM stage I tumors were considered as early-stage disease. As a result, the OS of the two gp130 expression sets was significantly different in diverse stage diseases. Specifically, the patients suffering from late-stage gastric cancer which expressed high gp130 had apparently poorer OS than those with low gp130 expression disease of the same stage did (Fig. 2e, *P* < 0.001). In contrast, no significance could be observed in terms of the OS between the two gp130 expression sets in TNM I gastric cancer (Fig. 2c, *P* = 0.0715). The DFS was statistically significant in both stages (Fig. 2d,f, *P* = 0.0077 and *P* < 0.001, respectively).

### Gp130 expression and benefit from 5-FU based adjuvant chemotherapy

**Figure 3:**
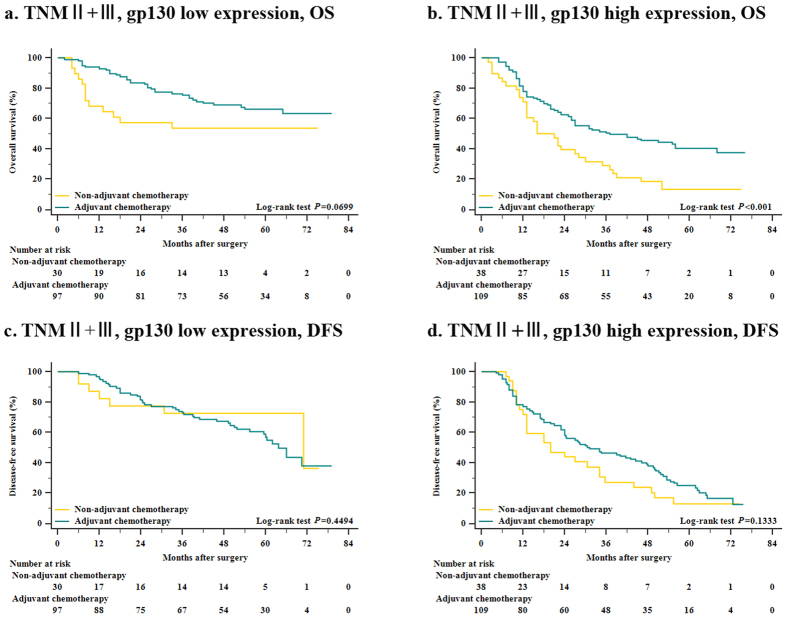
Relationship between gp130 expression and benefit from adjuvant chemotherapy. (**a**) In TNM stage II + III (advanced-stage) disease, no significant difference was observed in gp130-low subgroup (n = 127) when patients were given adjuvant chemotherapy (n = 97) or not (n = 30) (76.4% vs. 23.6%, *P* = 0.0699). (**b**) The rate of overall survival was significantly higher among the 109 patients with TNM stage II + III gp130-high tumors who were treated with adjuvant chemotherapy than among 38 patients who were not treated with adjuvant chemotherapy (74.1% vs. 25.9%, *P* < 0.001). (**c**) In TNM stage II + III disease, gp130-low subgroup (n = 127) did not show statistically significant difference in terms of receiving adjuvant chemotherapy or not (*P* = 0.4494). (**d**) However, in gp130-high cohort (n = 147), a non-significant trend (*P* = 0.1333) was observed towards a better disease-free survival among 109 patients who received adjuvant chemotherapy compared with the rest 38 not receiving adjuvant chemotherapy.

Furthermore, to assess whether patients with gp130-high tumors could benefit from 5-FU based adjuvant chemotherapy, we further investigated the correlation between gp130 expression, overall survival and disease-free survival among patients who either received adjuvant chemotherapy or not. Among the whole 370 patient population, 216 (58.4%) patients received chemotherapy and the relation between chemotherapy and gp130 expression was analyzed in Supplementary Table 1. Since TNM stage II or III patients generally require more aggressive postoperative treatment compared with stage I patients, we adjusted the patient population and focused on the stage II or III patients and excluded TNM stage I patients in our investigation of chemotherapy influence and correlation with gp130 expression. A preliminary test involving cohorts of patients with late-stage (TNM stage II and III) disease suggested a strong association between the use of adjuvant chemotherapy and a higher rate of OS in the stage II or III, gp130-high subgroup (Fig. 3b, *P* < 0.001), compared with the stage II or III, gp130-low subgroup (Fig. 3a, *P* = 0.0699). However, in late-stage disease, neither gp130-low subgroup nor gp130-high subgroup showed statistical significance in the DFS with respect to the utility of adjuvant chemotherapy (Fig. 3c,d, *P* = 0.4494 and *P* = 0.1333, respectively), regardless of a non-significant trend found in the gp-130 high subgroup. Hence, the results confirmed that postoperative treatment with 5-FU based adjuvant chemotherapy was closely associated with a higher rate of OS in the stage II or III, gp130-high patient population.

### Cox proportional hazards regression analysis and improvement of prognostic model on the basis of gp130 expression

**Figure 4:**
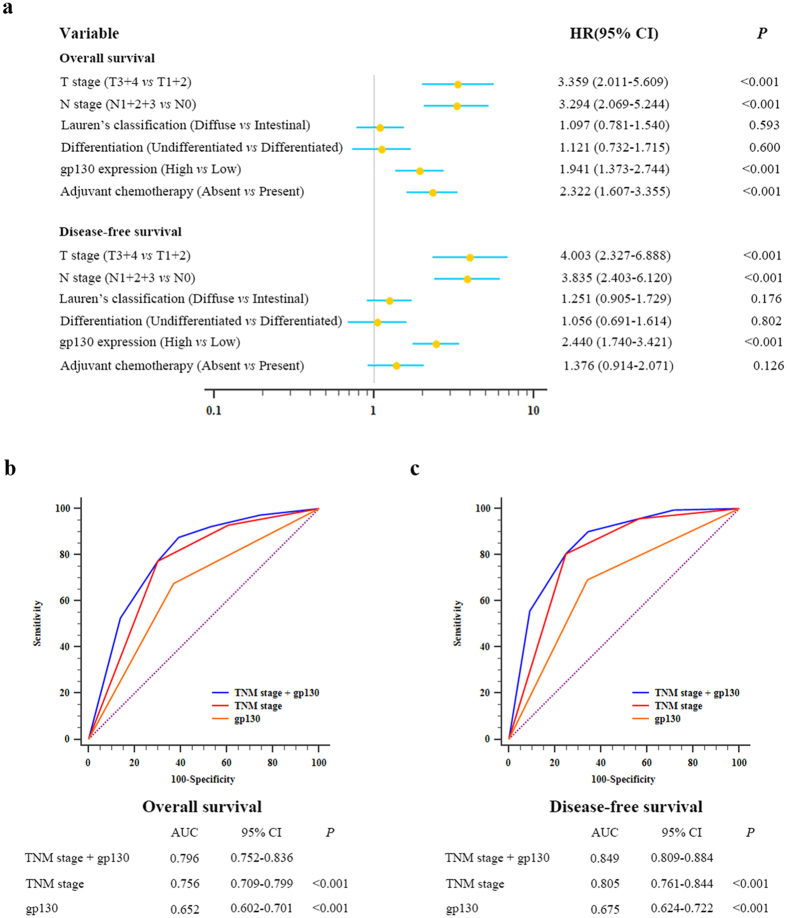
Cox multivariate analysis for independent prognostic factors and ROC analysis for the prognosis of OS and DFS. (**a**) Cox multivariate analysis identified the independent prognostic factors for OS and DFS for patients with non-metastatic gastric cancer. ROC analysis of the sensitivity and specificity for the prognosis of overall survival (**b**) and disease-free survival (**c**) by TNM stage/gp130 expression model, TNM stage model, and gp130 expression model.

Multivariate analysis revealed that intratumoral gp130 expression was a risk factor for OS, independent of tumor invasion, lymph node metastasis, and adjuvant chemotherapy. As to DFS, intratumoral gp130 expression served as a risk factor independent of tumor invasion as well as lymph node metastasis (Fig. 4a). In order to develop a more precise prognostic system, we consequently constructed a prognostic model, which combined the two independent prognostic factors, intratumoral gp130 expression and TNM stage. Area under the ROC curve (AUC) was applied to show the prognostic accuracy between different models. As a result, the association of gp130 expression with the TNM stage (AUC (95% CI), 0.796 (0.752–0.836) and 0.849 (0.809–0.884), respectively) showed more reliable prognostic effect than TNM stage (AUC (95% CI), 0.756 (0.709–0.799) and 0.805 (0.761–0.844), respectively) or gp130 expression (AUC (95% CI), 0.652 (0.602–0.701) and 0.675 (0.624–0.722), respectively) alone in both OS and DFS (Fig. 4b,c).

### Prognostic nomogram of 3-yr and 5-yr OS for non-metastatic gastric cancer

**Figure 5:**
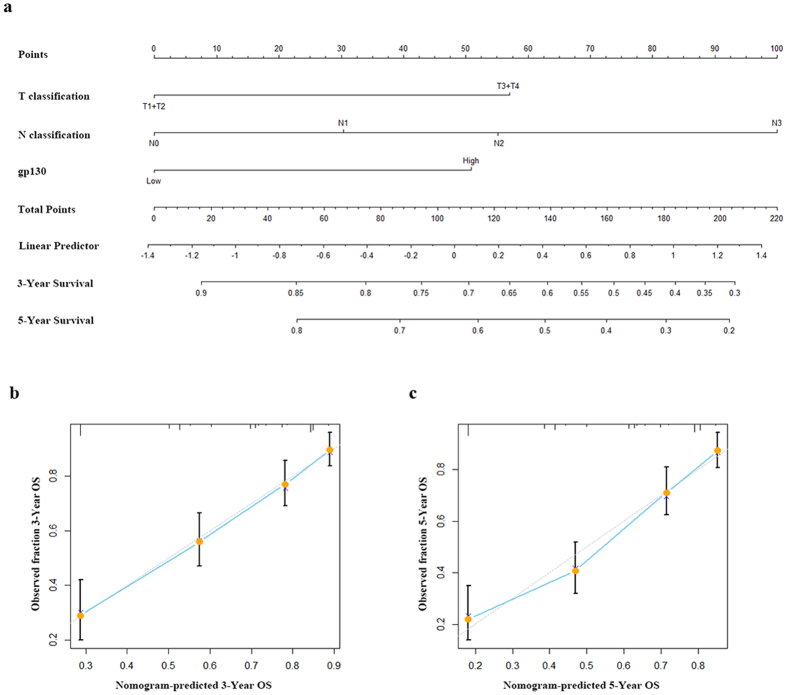
Nomogram and calibration plots for the prediction of outcomes in patients with non-metastatic gastric cancer. (**a**) Nomogram to predict overall survival (OS) at 3 years and 5 years after gastrectomy. (**b**) Calibration plot for nomogram predicted and observed 3-year overall survival rate. (**c**) Calibration plot for nomogram predicted and observed 5-year overall survival rate. Calibration curves for nomogram predicted 3 and 5-year overall survival performed pretty well with the ideal model. *Line of dashes*: ideal model; *vertical bars*, 95% confidence interval.

Since patients suffering from late-stage, gp130 high gastric cancer was observed to benefit from postoperative 5-FU based adjuvant chemotherapy, it shed light to a more suitable and individual treatment strategy based on the prognosis of every single non-metastatic gastric cancer patient. Consequently, a nomogram was further established to predict OS at 3 and 5 years after gastrectomy. The nomogram was built on the basis of the results from multivariate analysis of OS (Fig. 5a) with adjuvant chemotherapy excluded. All of the predictors, including T classification, N classification and gp130 expression, were not only independent prognostic indicators derived from multivariate analysis but also statistically significant to OS. In addition, the calibration plots of the nomogram were established to predict both 3-year and 5-year OS (Fig. 5b,c). Calibration curves for nomogram predicted 3-year and 5-year OS performed pretty well with the ideal model.

Ultimately, Harrell’s concordance index (C-index) and Akaike information criterion (AIC) were calculated to validate the result of nomogram. The C-index was 0.6867 and 0.6090 assessed with TNM stage and gp130 expression respectively but it significantly increased to 0.7161 when gp130 expression was combined with TNM staging system. Parallel to C-index, the AIC of TNM stage and gp130 expression was 1649.123 and 1704.127 respectively but decreased to 1636.015 if we incorporated gp130 expression into the current TNM staging system.

Conclusively, all of these results demonstrated that the association of gp130 expression with TNM staging system was able to generate a much more reliable prognostic model for OS prediction in patients with non-metastatic gastric cancer.

## Discussion

To our knowledge, so far no study has elucidated the clinical significance of gp130 in non-metastatic gastric cancer. Previous studies proved that gp130, serving as an inflammation-related cytokine receptor, plays an important part in the progression of several cancers such as ovarian cancer^16^, breast cancer^17^, prostate cancer^18^, colon cancer^19^ and lung cancer^20^, of which the mechanism for tumor progression is almost all related to IL-6 or IL-11/gp130/STAT3 signaling axis. STAT3 is recognized as the major signal transducer in the downstream of gp130 signaling pathway because STAT3 functions as an oncogene and is a key player which binds inflammation and cancer together^21^,^22^. Especially, chronic STAT3 activation was reported to serve as a key promoter in the induction and progression of gastric cancer. And it was consequently suggested that in chronic inflammation and metaplasia of gastric epithelium, targeting STAT3 or gp130 signal transduction might be therapeutically effective with regard to preventing gastric carcinogenesis^23^. It has also been demonstrated that in cellular models, STAT activation can result in increased cell survival and even escape from differentiation^24^. Fortunately, despite the crucial role which STAT activation plays in tumor development and progression, 5-FU could significantly decrease STAT activation in cancer cells and indicated a better effect than gemcitabine according to a previous breast cancer research^25^. 5-FU is an antimetabolite drug and functions its anti-tumor capability through blocking several essential biosynthetic processes, or incorporating itself into macromolecules as RNA or DNA to inhibit their normal biological function^26^. With great interest to see whether 5-FU has a similar impact on gastric cancer, we further investigated its therapeutic effect in our postoperative adjuvant chemotherapy research.

In our current study, patients stratified as late-stage gastric cancer victims were the candidates recruited in our 5-FU based postoperative adjuvant chemotherapy research. It is necessary to identify the subgroup patients whose tumor will not only be sensitive to 5-FU based adjuvant chemotherapy, but can lead to a higher rate of OS. Hence, we further evaluated the correlation between adjuvant chemotherapy and the clinical outcomes in patients with different gp130 expression tumors. As a result, in gp130-high patients, receiving adjuvant chemotherapy significantly resulted in a higher OS rate compared with non-adjuvant chemotherapy cohort, which suggested that gp130 could be a reliable factor in predicting the effectiveness of postoperative chemotherapy. Additionally, a trend to more satisfactory DFS rate in gp130-high, adjuvant chemotherapy-receiving patients was also observed although it was not statistically significant. This will be of great use for better selection and treatment of patients who should be recommended for adjuvant chemotherapy. However, our study is retrospective and the number of adjuvant chemotherapy-receiving patients is relatively small, and these results require validation in a prospective, larger, multi-centered randomized trial.

Conclusively, our study identified that elevated intratumoral gp130 expression was associated with unfavorable survival outcomes for gastric cancer patients and could be applied as an independent prognosticator. Combination of gp130 with current TNM staging system could lead to a more precise predictive model to further stratify patients with distinct prognosis. The findings also paved the way for individualized postoperative adjuvant chemotherapy based on different intratumoral gp130 expression, in that TNM II/III, gp130-high gastric cancer patients could significantly benefit from 5-FU based adjuvant chemotherapy. Detection of intratumoral gp130 expression might consequently help clinicians to supply more suitable clinical treatment and post-operative management strategy to gastric cancer patients.

## Methods

### Patients and specimens

A quantity of 370 non-metastatic gastric cancer patients who received radical gastrectomy with standard D2 lymphadenectomy between August 2007 and December 2008 in Zhongshan Hospital, Fudan University (Shanghai, China) was recruited in the current research. Among the whole cohort, 9 patients suffered from T3, N3, diffuse-type gastric cancer. No peritoneal dissemination was observed during the surgery with respect to these 9 patients. All patients enrolled in our study were informed of the consent which told the usage of human gastric cancer specimens. The consent was approved by the Clinical Research Ethics Committee of Zhongshan Hospital. The clinicopathological characteristics were retrospectively collected from each patient. Two independent gastroenterology pathologists respectively evaluated the specimens according to the 2010 International Union Against Cancer TNM classification system. Both of the gastroenterology pathologists were blind to patients’ clinicopathological data. 5-FU based postsurgical adjuvant chemotherapy was applied for the patients diagnosed with advanced-stage gastric cancer or early-stage cancer with aggressive lymph node metastasis. Collectively, there were 216 (58.4%) patients who received 5-FU based ACT in the whole cohort. The main endpoint of interest was overall survival (OS) and secondary end point was disease-free survival (DFS), respectively. OS and DFS were defined as the period of time from the date of surgery to the date of death and disease recurrence, respectively, or to the date of the last follow-up. Patients were observed until April 2014. The length of our follow-up time was from 2 months to 79 months, and the median follow-up time was 49 months. According to our follow-up, 216 of 370 (58.4%) patients were alive whereas 154 (41.6%) were dead with respect to overall survival. As to disease-free survival, 205 of 370 (55.4%) stayed disease-free while 165 (44.6%) suffered from disease recurrence respectively. All methods were approved by the Clinical Research Ethics Committee of Zhongshan Hospital, Fudan University and were carried out in accordance with the approved guidelines. Written informed consent on the use of specimens from each patient was achieved.

### Cell lines, protein extraction and western blot analysis

Human gastric cancer cell lines HGC-027, HGC-803, HGC-7901 and AGS were directly obtained from Shanghai Cell Bank of Chinese Academy of Sciences (Shanghai, China). HGC-027 cell lines were cultured in RPMI medium 1640 and HGC-803, HGC-7901 and AGS were cultured in Dulbecco’s modified Eagle’s medium (DMEM), respectively. The RPMI medium 1640 and DMEM were both supplemented with 10% fetal bovine serum in a humidified 5% CO_2_ incubator at 37 °C.

Protein extraction from cultured cell lines and consequent western blot analysis were performed as described elsewhere^27^. In brief, cultured cells were washed with phosphate buffered saline (PBS) and then lysed with use of lysis buffer (10 mM Tris–HCl, 1% Triton-X 100, 150 mM NaCl, aprotinin, leupeptin and 1 mM phenylmethylsulfonyl fluoride). Insoluble materials were eliminated by means of 15,000 rpm centrifugation for 10 min.

Equal quantities of protein were separated with use of 7.5% sodium dodecyl sulfate–polyacrylamide gel electrophoresis (SDS–PAGE), and were transferred to a PVDF membrane (Millipore, Billerica, MA). Then, the PVDF membranes were blocked with 5% non-fat dry milk, incubated with primary antibody against gp130 (ab202850, Abcam, Cambridge, MA, USA) and GAPDH (Santa Cruz Biotechnology, Santa Cruz, CA) overnight at 4 °C and subsequently reacted with horseradish peroxidase-conjugated secondary antibodies (sc-2004 and sc-2005 respectively, Santa Cruz Biotechnology) for 2 hours at room temperature. Bands were visualized with use of the ECL detection system (GE Healthcare, Chalfont St Giles, UK).

### Tissue microarray and immunohistochemistry

Formalin-fixed, paraffin-embedded gastrectomy specimens were used for tissue microarrays (TMA) establishment and the following immunohistochemistry (IHC) study. The tissue microarrays were performed according to the protocol previously described^28^. Briefly, two tissue cores for a patient were taken from each representative tumor tissue and from gastric tissue adjacent to the tumor within a distance of 5 cm to construct TMA slides. Before the establishment of tissue microarrays, 4-μm thick sections were sliced from each tissue block and then stained with use of Hematoxylin & Eosin (H&E) for the confirmation of diagnosis and the selection of representative area for each tissue sample. Next, tissue cylinders of 2 mm in diameter containing either tumor tissues or non-cancerous gastric tissues were punched out from the chosen area of each tissue block and then transferred into a TMA block by means of a TMA instrument. Subsequently, 4-μm thick sections were consecutively sliced from each TMA block and one of the sections was specially stained with H&E for histological validation in order to make sure the arrayed gastric tumor tissue samples were adequately constructed. Qualified gastric tumor tissue samples were identified as the samples in which the tumor tissue accounted for over 10% of the core area. Sections were subsequently placed on microscope slides, waiting for immunohistochemistry.

Immunohistochemistry for gp130 was performed according to the protocol described elsewhere^29^. During the immunohistochemistry, the time and temperature were strictly controlled for every single tissue microarray. The whole set of tissue specimens was also processed and immunostained at the same time so as to ensure an objective comparison between different samples. The tissue microarrays were baked at 60 °C for 6 hours, deparaffinized in xylene and rehydrated in graded alcohol at room temperature. Endogenous peroxidase was blocked with use of 3% H_2_O_2_ in methanol at 37 °C for 20 minutes. Then, the microarrays were immersed in 0.01 M citrate buffer (pH 6.0) and cooked for antigen retrieval. After cooling down to the room temperature, the microarrays were subsequently incubated with 10% normal goat serum at 37 °C for 30 minutes in order to eliminate nonspecific reactions. The primary antibody against gp130 (1:400 dilution, anti-CD130 (gp130) antibody ab202850, Abcam, Cambridge, MA, USA) was applied. Negative controls were treated identically but with the primary antibody omitted. The microarrays were incubated with anti-gp130 antibody at 4 °C overnight. Being rinsed three times in 0.01 M phosphate buffer (pH = 8.0), the microarrays were then incubated with secondary antibody at 37 °C for 20 minutes and stained with diaminobenzidine (DAB)-H_2_O_2_ for 2 minutes. Finally, the microarrays were counterstained with hematoxylin, dehydrated and mounted.

### Evaluation of Immunostaining

The immunostaining was evaluated under a light microscope by 2 pathologists who were blind to the clinical data and scored independently according to the intensity of cellular staining and the proportion of stained tumor cells. The IHC staining assessment was constructed according to the protocol previously described as well^30^. Briefly, the staining degree was stratified into 4 categories as 0 (negative staining), 1 (weak staining, light yellow), 2 (moderate staining, yellow), and 3 (strong staining, brown), while the proportion of stained tumor cells was graded as the percentage of positive cells (0–100%). The IHC staining intensity score ranging from 0–300 was generated after the combination of staining degree and positive tumor cell proportion by multiplication. All tissue microarrays were assessed independently and paired at the end. In cases of discrepancy, if the variability between the two scores of a certain tumor tissue sample was more than 5%, then the tumor tissue sample would be reviewed to reach the final consensus score, or we chose an average value of the two discordant scores. The cut-off value for the definition of high/low gp130 expression subgroups was the median value.

### Statistical analysis

Pearson’s chi-squared test or Fisher’s exact test was applied for categorical variables. Student’s *t* test was applied for the analysis of continuous variables. Survival curves were established on the basis of the Kaplan–Meier methodology and the significance of differences between survival curves was calculated by means of the log-rank test. Multivariate analysis was constructed through the Cox proportional hazards regression model. Statistical analysis was performed with the help of SPSS Statistics 21.0 (SPSS Inc., Chicago, IL). The nomogram analysis and calibration plot were produced via the R software version 3.0.2 and the ‘rms’ package (R Foundation for Statistical Computing, Vienna, Austria). Harrell’s index of concordance (C-index) and Akaike information criterion (AIC) were both calculated in order to compare and validate the accuracy of the predictive models. All statistical tests were two sided and *P* < 0.05 was considered statistically significant.

## Additional Information

**How to cite this article**: Cao, Y. *et al*. Glycoprotein 130 is associated with adverse postoperative clinical outcomes of patients with late-stage non-metastatic gastric cancer. *Sci. Rep.* **6**, 38364; doi: 10.1038/srep38364 (2016).

**Publisher's note:** Springer Nature remains neutral with regard to jurisdictional claims in published maps and institutional affiliations.

## Supplementary Material

Supplementary Information

## Figures and Tables

**Table 1:** Relationship between gp130 expression and clinical characteristics.

Factors	Patients		gp130 expression		
	No.	%	Low	High	*P*-value
All patients	370	100	186	184	
Age (years)^a^					0.077
<60	180	48.6	99	81	
≥60	190	51.4	87	103	
Gender					0.963
Male	261	70.5	131	130	
Female	109	29.5	55	54	
Tumor size (cm)					0.200
Mean ± SD	3.8 ± 2.2		3.6 ± 2.0	3.9 ± 2.2	
Differentiation					0.529
Differentiated	104	28.1	55	49	
Undifferentiated	266	71.9	131	135	
Lauren classification					0.293
Intestinal type	235	63.5	123	112	
Diffuse type	135	36.5	63	72	
T classification					**0.003**
T1	70	18.9	42	28	
T2	58	15.7	34	24	
T3	67	18.1	40	27	
T4	175	47.3	70	105	
N classification					**<0.001**
N0	149	40.3	90	59	
N1	43	11.6	24	19	
N2	67	18.1	35	32	
N3	111	30	37	74	
TNM stage					**<0.001**
I	96	26.0	59	37	
II	90	24.3	54	36	
III	184	49.7	73	111	
Adjuvant chemotherapy^b^					0.165
Yes	216	58.4	102	114	
No	154	41.6	84	70	

gp130 = glycoprotein 130; TNM = tumor node metastasis; *P*-value < 0.05 marked in bold font shows statistical significance.

^a^Split at median.

^b^Patients with adjuvant chemotherapy received at least one cycle of 5-fluoruracil based chemotherapy.
