# Supplementary material for: Glycoprotein 130 is associated with adverse postoperative clinical outcomes of patients with late-stage non-metastatic gastric cancer
Source: Sci Rep. 2016 Dec 5;6:38364. doi: 10.1038/srep38364 (PMC5137155; doi:10.1038/srep38364)
Supplement: Supplementary Information [file srep38364-s1.doc]

**Glycoprotein 130 is associated with adverse postoperative clinical outcomes of patients with late-stage non-metastatic gastric cancer**

Yifan Cao^1,3^, Heng Zhang^2,3^, Hao Liu^2,3^, Chao Lin^2^, Ruochen Li^1^, Songyang Wu^1^, Hongyong He^2,*^, He Li^2,*^, Jiejie Xu^1,*^

^1^Department of Biochemistry and Molecular Biology, School of Basic Medical Sciences, Fudan University, Shanghai, China;

^2^Department of General Surgery, Zhongshan Hospital, Fudan University, Shanghai, China;

^3^These authors contributed equally to this work.

***Corresponding authors.** **Hongyong He,** Department of General Surgery, Zhongshan Hospital, Fudan University, Shanghai 200032, China. Tel: +86 21 64041990-2910; Fax: +86 21 64038472; E-mail: [he.hongyong@zs-hospital.sh.cn](mailto:he.hongyong@zs-hospital.sh.cn); **He Li,** Department of General Surgery, Zhongshan Hospital, Fudan University, Shanghai 200032, China. Tel: +86 21 64041990-2910; Fax: +86 21 64038472; E-mail: [li.he@zs-hospital.sh.cn](mailto:li.he@zs-hospital.sh.cn); or **Jiejie Xu**, Department of Biochemistry and Molecular Biology, School of Basic Medical Sciences, Fudan University, Shanghai 200032, China. Tel: +86 21 54237332; Fax: +86 21 64437703; E-mail: [jjxufdu@fudan.edu.cn](mailto:jjxufdu@fudan.edu.cn).


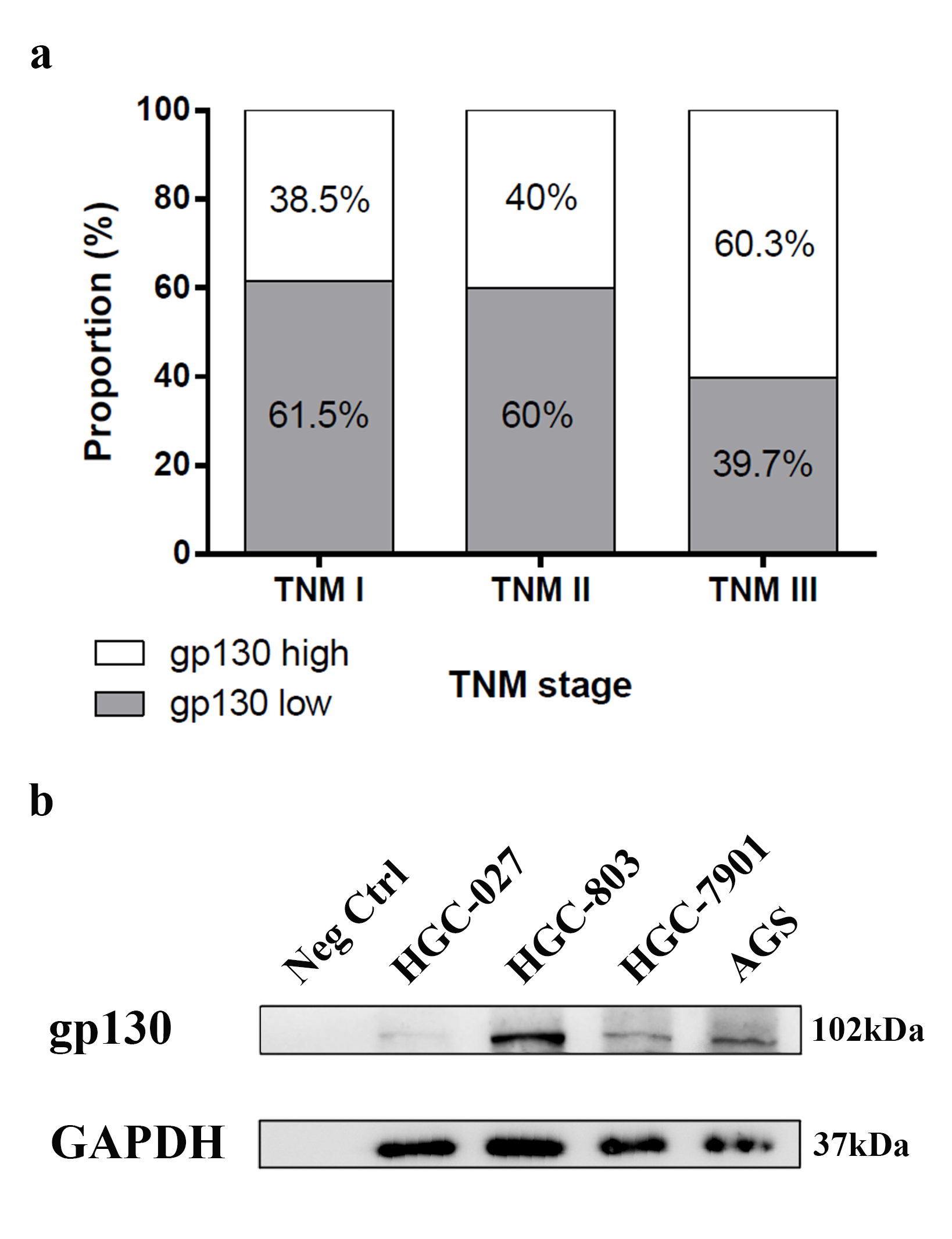


**Supplementary Figure 1. Supplementary information about gp130. (a)** The percentage of patients with high or low immunohistochemistry stained intratumoral gp130 expression was compared on the basis of the TNM stage. **(b)** Western blot analysis was applied to detect the expression of gp130 and GAPDH in HGC-027, HGC-803, HGC-7901 and AGS cells, respectively. The diversity of gp130 expression in different gastric cancer cell lines was observed, which indicated the specificity of anti-gp130 antibody as well.

Supplementary Table 1. The influence of chemotherapy on patients and its relation with gp130 expression.

| **Factors** |  | | **Overall survival** | | **Disease-free survival** | |
| --- | --- | --- | --- | --- | --- | --- |
|  | **No.** | **%** | **Hazard ratio (95% Cl)** | ***P*-value** | **Hazard ratio (95% Cl)** | ***P*-value** |
| All patients | 370 | 100 |  | 0.247 |  | **<0.001** |
| ACT | 216 | 58.4 | 1.00(reference) |  | 1.00(reference) |  |
| Non-ACT | 154 | 41.6 | 0.825(0.598-1.138) |  | 0.393(0.289-0.535) |  |
| gp130-low patients | 186 | 100 |  | 0.168 |  | **<0.001** |
| ACT | 102 | 54.8 | 1.00(reference) |  | 1.00(reference) |  |
| Non-ACT | 84 | 45.2 | 0.666(0.381-1.164) |  | 0.221(0.128-0.384) |  |
| gp130-high patients | 184 | 100 |  | 0.942 |  | **0.024** |
| ACT | 114 | 62.0 | 1.00(reference) |  | 1.00(reference) |  |
| Non-ACT | 70 | 38.0 | 0.986(0.663-1.465) |  | 0.533(0.367-0.775) |  |
| Adjusted^a^ patients | 274 | 100 |  | **<0.001** |  | 0.373 |
| ACT | 206 | 75.2 | 1.00(reference) |  | 1.00(reference) |  |
| Non-ACT | 68 | 24.8 | 2.008(1.323-3.047) |  | 1.187(0.796-1.769) |  |
| Adjusted gp130-low patients  ACT  Non-ACT | 127  97  30 | 100  76.4  23.6 | 1.00(reference)  1.788(0.844-3.791) | 0.070 | 1.00(reference)  0.736(0.359-1.513) | 0.449 |
| Adjusted gp130-high patients  ACT  Non-ACT | 147  109  38 | 100  74.1  25.9 | 1.00(reference)  2.006(1.224-3.286) | **<0.001** | 1.00(reference)  1.386(0.860-2.234) | 0.133 |
| Abbreviations: CI= confidence interval; gp130= glycoprotein 130; ACT= adjuvant chemotherapy; TNM = tumor-node-metastasis. *P*-value < 0.05 marked in bold font shows statistical significance.  ^a^“Adjusted” refers to the adjusted patient population in which TNM stage I patients were excluded and only stage II or III patients were included. | | | | | | |
